# SO2426 is a positive regulator of siderophore expression in *Shewanella oneidensis *MR-1

**DOI:** 10.1186/1471-2180-11-125

**Published:** 2011-05-31

**Authors:** Kristene L Henne, Xiu-Feng Wan, Wei Wei, Dorothea K Thompson

**Affiliations:** 1 Department of Biological Sciences, Purdue University, West Lafayette, IN 47907, USA; 2 Department of Basic Sciences, College of Veterinary Medicine, Mississippi State University, Mississippi State, MS 39762, USA; 3 Center for Environmental Biotechnology, University of Tennessee, Knoxville, TN 37996, USA

## Abstract

**Background:**

The *Shewanella oneidensis *MR-1 genome encodes a predicted orphan DNA-binding response regulator, SO2426. Previous studies with a SO2426-deficient MR-1 strain suggested a putative functional role for SO2426 in the regulation of iron acquisition genes, in particular, the siderophore (hydroxamate) biosynthesis operon *so3030*-*3031*-*3032*. To further investigate the functional role of SO2426 in iron homeostasis, we employed computational strategies to identify putative gene targets of SO2426 regulation and biochemical approaches to validate the participation of SO2426 in the control of siderophore biosynthesis in *S. oneidensis *MR-1.

**Results:**

*In silico *prediction analyses revealed a single 14-bp consensus motif consisting of two tandem conserved pentamers (5'-CAAAA-3') in the upstream regulatory regions of 46 genes, which were shown previously to be significantly down-regulated in a *so2426 *deletion mutant. These genes included *so3030 *and *so3032*, members of an annotated siderophore biosynthetic operon in MR-1. Electrophoretic mobility shift assays demonstrated that the SO2426 protein binds to its motif in the operator region of *so3030*. A "short" form of SO2426, beginning with a methionine at position 11 (M11) of the originally annotated coding sequence for SO2426, was also functional in binding to its consensus motif, confirming previous 5' RACE results that suggested that amino acid M11 is the actual translation start codon for SO2426. Alignment of SO2426 orthologs from all sequenced *Shewanella *spp. showed a high degree of sequence conservation beginning at M11, in addition to conservation of a putative aspartyl phosphorylation residue and the helix-turn-helix (HTH) DNA-binding domain. Finally, the *so2426 *deletion mutant was unable to synthesize siderophores at wild-type rates upon exposure to the iron chelator 2,2'-dipyridyl.

**Conclusions:**

Collectively, these data support the functional characterization of SO2426 as a positive regulator of siderophore-mediated iron acquisition and provide the first insight into a coordinate program of multiple regulatory schemes controlling iron homeostasis in *S. oneidensis *MR-1.

## Background

Bacteria sense and respond to environmental stimuli primarily through signal transduction pathways, in which the canonical mechanism employs a sensor histidine kinase that interacts with a DNA-binding response regulator to activate or repress specific gene transcription [1,2]. Some cellular processes have been shown to be controlled by orphan response regulators or one-component signalling systems, in which a cognate sensor kinase has not been elucidated [3]. Orphan response regulators have been shown to be involved in the regulation of motility and chemotaxis [4], growth-phase-dependent responses [5,6], virulence [7], iron transport [8] and oxidative stress responses [8,9]. For instance, one well-characterized regulon that appears to be controlled by an orphan response regulator in *S. oneidensis *MR-1 is the ArcA regulon, which regulates the cellular response to aerobic and anaerobic respiratory conditions [10]. The distinguishing feature of ArcA in comparison to the analogous system in *Escherichia coli *is that there does not seem to be a cognate sensor kinase, ArcB, in *S. oneidensis *[10], suggesting that *S. oneidensis *ArcA may be an orphan response regulator.

Our previous work suggested that a putative orphan response regulator, SO2426, in *S. oneidensis *MR-1 may be an integral member of a metal-responsive regulon governing the up-regulation of genes involved in iron uptake and homeostasis in response to metal stress. The ferric iron uptake regulator (Fur) is the predominant mechanism by which bacteria regulate iron homeostasis [11]. Evidence suggests an additional iron responsive network regulated by SO2426 in *S. oneidensis *MR-1. Up-regulation of SO2426 at both the protein and transcript levels in response to iron and acid stress has been observed in a Δ*fur *mutant strain of MR-1 [12-14]. Our previous studies investigating the transcriptomic and proteomic response of *S. oneidensis *to chromate challenge further revealed enhanced expression of *so2426 *under chromate stress [15,16]. In a *so2426 *deletion mutant, genes involved in iron acquisition and homeostasis such as the *so3030*-*3031*-*3032 *operon, which encodes siderophore biosynthesis genes, were consistently down-regulated at high levels in the deletion mutant. Iron acquisition and storage systems are commonly up-regulated when bacteria are subjected to conditions of metal stress (*e.g*., chromate), and a link between iron transport and heavy metal sensitivity has been suggested [15,17]. It is possible that sequestration of iron prevents redox cycling between ferrous iron and chromate, which can lead to reactive intermediates and oxidative stress [18,19]. A consequence of this may be deficient intracellular iron concentrations that could inhibit growth. A cyclical response would ensue, resulting in up-regulation of iron uptake genes such as those involved in siderophore biosynthesis, which is similar to what has been demonstrated for *S. oneidensis *in response to chromate stress [15,16,20].

The aim of the present study was to examine the function of the uncharacterized SO2426 response regulator within the context of siderophore biosynthesis. We used a bioinformatics approach to map putative SO2426-binding domains and biochemical assays to demonstrate the binding of SO2426 to predicted recognition sites. Electrophoretic mobility shift assays showed that a recombinant SO2426 protein binds to a putative SO2426 motif that exists within the operator region of the *so3030*-*3031*-*3032 *operon. Siderophore detection assays further showed a diminished capacity of the Δ*so2426 *mutant strain to produce siderophores, particularly in the presence of the iron chelator 2,2'-dipyridyl. Based on the identification of a Fur-binding motif upstream of the predicted SO2426-binding site within the operator region of the *so3030*-*3031*-*3032 *operon, we postulate that there are likely multiple levels of regulation operating in *S. oneidensis *MR-1 to precisely adjust intracellular iron levels in response to cellular needs. These intricate control mechanisms appear to involve Fur-mediated repression and derepression as well as SO2426-mediated activation of siderophore biosynthesis genes.

## Results and Discussion

### Conservation of SO2426 amino acid sequence among *Shewanellae*

**Figure 1:**
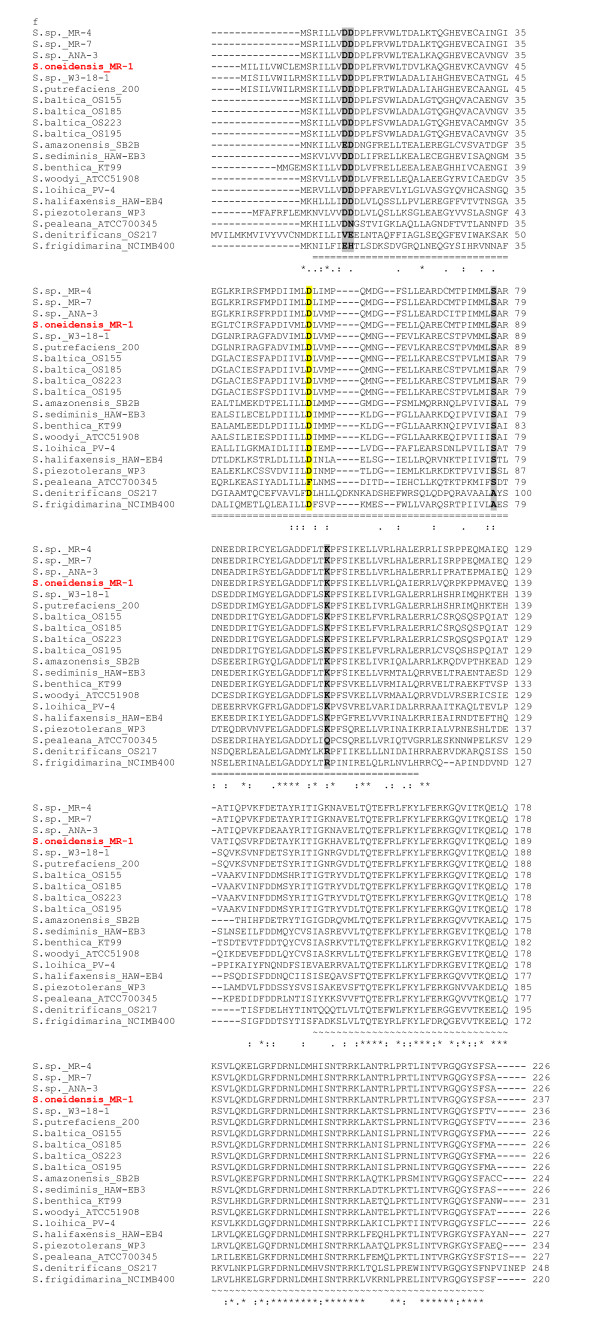
**Sequence alignment of SO2426 orthologs from sequenced *Shewanella *species**. ClustalW was used to perform a multiple sequence alignment of *Shewanella *SO2426 orthologs. The region underlined with "=" is the aligned regulator receiver domain with predicted domain (SO2426: positions 13-124), and the region denoted with "~" is the aligned C-terminal domain containing the wHTH DNA-binding motif (SO2426: positions 158-235). Boldface letters highlighted in grey indicate conserved signature residues of receiver domains. Residue D62 is predicted as 4-aspartylphosphate, the putative phosphorylation site (highlighted in yellow). The star, colon, and dot notations rank the sequence conservation from high to low, respectively. The GenBank accession numbers and associated *Shewanella *species are provided in the Methods.

Previously, we reported that the *so2426 *gene of *S. oneidensis *MR-1 shares 27 to 36% sequence identity at the amino acid level to CpxR and OmpR orthologs from *Vibrio cholerae *and *Escherichia coli *[21]. Orthologs of SO2426 were also identified in a number of *Shewanella *species. Multiple sequence alignment of all available *Shewanella *SO2426 orthologs revealed a high degree of conservation at key residues (Figure 1). The predicted phosphorylation residues (D18, D19, D62, and K109) associated with the N-terminal CheY-like response regulator domain of SO2426 [21] are highly conserved among *Shewanella *orthologs. Another striking feature is the high degree of sequence conservation among the C-terminal or output domains of the SO2426 orthologs. This region contains several features of OmpR winged-helix transcriptional regulators such as the output domain, encompassed by residues T225, G230, and Y231 [22]. Residues 204-215 (LDMHISNTRRKL) resemble the predicted α3-helical region of *E. coli *CpxR, which comprises the crucial recognition sequence of OmpR-like response regulators [22]. Moreover, it is noteworthy that the annotated 5' terminus of the majority of sequenced *Shewanella *SO2426 orthologs occurs at M11 relative to the MR-1 sequence (Figure 1). Previous 5' RACE analysis of the transcription start site of MR-1 SO2426 demonstrated that M16 (or M11 relative to the MR-1 sequence) is likely the correct start residue [21].

**Figure 2:**
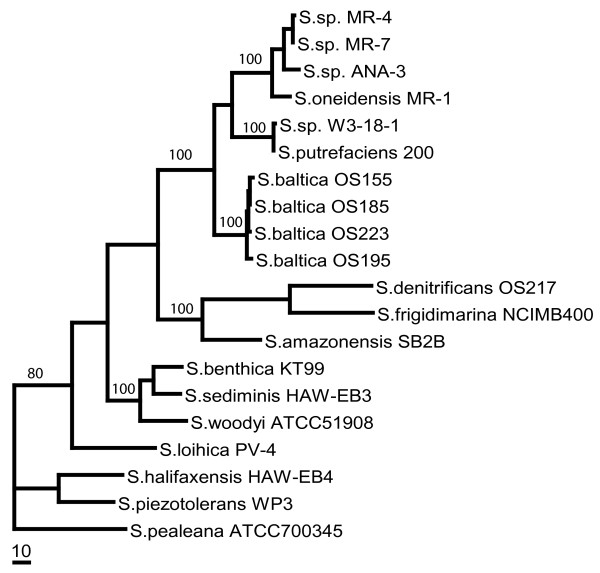
**Phylogenetic tree of SO2426 orthologs in *Shewanella *spp**. The phylogenetic tree was constructed based on protein sequences using the maximum parsimony method implemented in PAUP* version 4.0 Beta [54]. Bootstrap values were generated using maximum parsimony. The bar scale indicates a branch length corresponding to 10 character-state changes. The GenBank accession numbers are provided in the Methods.

A phylogenetic tree constructed from the multiple sequence alignment in Figure 1 shows that SO2426 clusters tightly with sequences from *Shewanella *spp. MR-4, MR-7, and ANA-3 (Figure 2). In a system-wide comparison of *Shewanella *species, it was recently shown that MR-1, MR-4, MR-7, and ANA-3 tend to be more closely related to each other than to other *Shewanellae *when comparing genomes, proteomes, gene content, and 16S rRNA sequences [23]. These four species exhibit physiological characteristics consistent with their ability to adapt to harsh environments, which is a hallmark characteristic of *Shewanella *[24]. Strain ANA-3 is most recognized for its ability to respire arsenate [25] but has also been shown to harbor a chromate efflux operon [26], and like MR-1, MR-4 is a known chromate reducer [27]. Synteny of other gene clusters among strains MR-1, MR-4, MR-7, and ANA-3 has been noted for other metabolic processes [28] and cytochrome operons associated with metal reduction [29]. Given the shared genetic and proteomic arrangements among these strains, it is likely that sequence-level relatedness will translate to shared phenotypic traits.

### *In silico *prediction of the SO2426 recognition site

**Figure 3:**
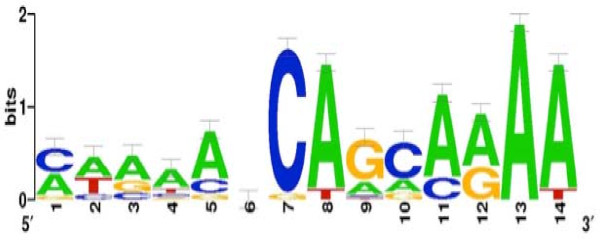
**Identification of a predicted consensus SO2426-binding motif in *S*. *oneidensis *MR-1 using computational methods**. A sequence logo representation [51] of a 14-bp motif model was derived using promoter regions directly upstream of 46 clustered genes exhibiting down-regulated expression in a Δ*so2426 *mutant strain of MR-1 [21]. The error bars indicate standard deviations.

Three complementary computer-based motif search tools--MEME [30], MotifSampler [31], and Gibbs Recursive Sampler [32]--were applied to microarray data generated for a Δ*so2426 *MR-1 mutant strain [21] in order to predict a consensus recognition site potentially bound by the SO2426 response regulator (see Methods for details). The computational analyses identified a single 14-bp consensus motif in the input dataset (Figure 3). This recognition weight matrix consisted of two conserved pentamers (5'-CAAAA-3') in tandem (with the first one being much less conserved), separated by the 4-bp linker sequence 5'-NCAG-3'. The linker sequence composition is not random in that positions 7 and 8 in the motif contain a well-conserved C and A residue, respectively (Figure 3). Other two-component response regulators that also recognize a tandem repeat sequence include phosphorylated CpxR (CpxR-P) and OmpR-P. The closest known homolog of *S*. *oneidensis *SO2426 is CpxR [21]. Intriguingly, the predicted SO2426 recognition sequence resembles the proposed CpxR binding box [5'-GTAAA-(N)_5_-GTAAA-3'] [33,34]. The MR-1 *cpxR *gene was down-regulated three-fold in *Δso2426 *mutant cells challenged with chromate [21] compared to a three-fold induction that was observed for wild-type MR-1 cells under similar conditions [15]. The CpxAR two-component system functions in responding to cell envelope stress and external environmental stimuli, leading to the activation of genes involved in repairing misfolded proteins [1,35,36]. The Cpx system has been implicated in a number of cellular responses including the activation of outer membrane porins [37], stationary phase-induced survival mechanisms [38], and pH stress [39]. Given the activation of CpxR orthologs such as SO2426 during periods of chromate stress in *S. oneidensis *MR-1 [15,21] and copper stress in *E. coli *[40], it is suspected that Cpx and analogous systems operate to overcome oxidative membrane and protein damage induced by exposure to toxic metal ions.

For the present study, we used an input dataset for SO2426 recognition site prediction consisting of 46 genes showing similar down-regulated temporal expression patterns in the Δ*so2426 *mutant [21]. As computational analysis showed, a number of these co-regulated genes were preceded by a conserved tandem repeat (5'-CAAAANCAGCAAAA-3') and included genes *so2280 *(a putative *bcr*), *so1188, so1190, so3025, so3062, ftn, so1580, so 2045, so3030, so3032, viuA*, and *so4743 *(see Table 1). The majority of these putative SO2426-binding sites were located in upstream regulatory regions, while one site was identified in the *so1190 *coding region near the annotated 5' terminus at position +12, suggesting that expression of the *so1188*-*1189*-*1190 *operon might be controlled by internal secondary *cis*-regulatory elements. In addition, two tandem 5'-CAAAA-3' motifs were identified upstream of the *so2426 *locus at position -88 relative to the annotated translation start codon (Table 1), pointing to the possible involvement of an autoregulatory mechanism. Interestingly, a subset of the genes repressed in the Δ*so2426 *mutant, namely genes with functions in iron acquisition and storage, also possessed a predicted ferric uptake regulator (Fur) box in their upstream regulatory regions. A potential Fur recognition motif, 5'-AAATGAtATTGATTcTCgTTT-3', was identified in the upstream region flanking *so2426 *and overlapped the transcriptional start sites for this gene [21].

**Table 1:** Putative SO2426 gene targets containing the predicted SO2426-binding site

ORF	Functional Category/Gene Product	Motif	Strand	Distance^*a*^	E-value^*b*^
	**Cellular processes**				
SO2280	bicyclomycin resistance protein	AACGCTCAGGCAAA	-	-241	2.06E-04
	**Central intermediary metabolism**				
	5-methylthioadenosine nucleosidase/S-				
SO3705	adenosylhomocysteine nucleosidase, putative	GTCAGCCAGCAAAA	+	+21	4.73E-05
	**Energy metabolism**				
SO2743	acetyl-coenzyme A synthetase (*acs*)	AAAAAAGAGCAAAA	-	-160	1.46E-05
	**Hypothetical proteins**				
SO1188	conserved hypothetical protein	AAAACTCAGCAGAA	-	-113	2.08E-06
SO1190	conserved hypothetical protein	CTAAGGCAACAAAA	-	+12	2.38E-05
SO1770	glycerate kinase, putative	ACAACCCAGAAGAA	-	-177	2.61E-05
SO3025	conserved hypothetical protein	GCAAAACATCAAAA	+	-234	1.13E-04
SO3062	hypothetical protein	ATAAATCAGGAGAA	+	-5	7.64E-06
SO4499	hypothetical protein	CTGCAACAGGAGAA	+	-5	1.19E-05
SO4504	conserved hypothetical protein	ATGTCCCAGACAAA	+	-169	1.06E-04
SO4719	conserved hypothetical protein	ATGAACCACAAGAA	+	-199	9.88E-05
	**Transport and binding proteins**				
SO0139	ferritin (*ftn*)	CAAAAGCAACAAAA	-	-63	2.08E-06
SO1580	TonB-dependent heme receptor	AAAAAGCAGAAAAA	-	-112	3.68E-06
SO1771	permease, GntP family	CTACAACAGCCAAA	+	-41	2.81E-06
SO2045	cation efflux family protein	CACCCTCAACAGAA	+	+11	5.98E-05
SO3030	siderophore biosynthesis protein (*alcA*)	CTGTAACAGCAAAT	+	-133	2.86E-05
SO3032	siderophore biosynthesis protein, putative	CCGGATCAGCAAAA	+	-284	1.46E-05
SO3033	ferric alcaligin siderophore receptor	ATCAAACAGCCAAA	+	-112	3.20E-06
SO3063	sodium:alanine symporter family protein	CAAAAACAACAGAA	+	-18	1.09E-06
SO4150	transporter, putative	AAAAAACTGCAGAA	+	+16	7.64E-06
SO4516	ferric vibriobactin receptor (*viuA*)	CAGTAGCAGAAGAA	+	-249	1.62E-05
SO4743	TonB-dependent receptor, putative	CAAAAACAACAAAT	-	-168	2.38E-05
	**Signal transduction**				
SO2426	DNA-binding response regulator	CAATACCTGCCAAA	+	-88	5.12E-05

^*a*^Distance in base pairs of the start of the potential SO2426 binding site from the first nucleotide of the predicted translation start codon of the corresponding gene listed in the first column. ^*b*^The E-value of the motif is an estimate of the number of motifs with the same width and number of occurrences that would have equal or higher log likelihood ratio if the training set sequences had been generated randomly according to the background model [30].

Several lines of evidence further support the role of *so2426 *in the regulation of iron acquisition in *S. oneidensis *MR-1. A recent study applying gene network reconstruction to MR-1 indicated that SO2426 clusters with iron acquisition genes in a distinct iron-responsive network system [14]. Within this iron acquisition gene network were a number of members of the SO2426 regulon proposed here, namely, *so1188, so1190, so3025, so3030*-*3031*-*3032, so3063*, and *so4743 *[14]. All of these genes, including *so2426*, were up-regulated under iron-depleted growth conditions compared to iron-replete conditions. Additionally, *so3030 *was up-regulated almost 14-fold in a *fur *mutant, while genes *so3031-so3033 *were up-regulated 4 to 11-fold [13]. A separate transcriptomic study with a *fur *deletion mutant revealed that the gene with the greatest expression change in the *fur *mutant compared to the MR-1 wild-type strain was *so2426*, which showed a 30- and 26-fold increase in expression at the transcript level under aerobic and anaerobic growth conditions, respectively [12]. In addition to the enhanced expression of *so2426 *in a *fur *mutant, this gene has been shown to be up-regulated under chromate [15,41] and strontium [42] stress.

The presence of a putative Fur-binding sequence in the promoter region of *so2426 *suggests that expression of this response regulator may be subject to multiple levels of regulatory control. Identification of a Fur box immediately downstream of the -10 promoter element and up-regulation of *so2426 *expression in a *fur *deletion mutant are both consistent with repression of this gene by Fur under iron-sufficient conditions. Similarly, of those genes encoding transport and binding proteins, *ftn, so1580*, the *so3030*-*3031*-*3032 *operon, *so4516*, and *so4743 *are probable members of the Fur regulon based on their derepressed expression patterns in a *S*. *oneidensis *Δ*fur *mutant and the presence of a putative Fur box in their respective upstream regions [12]. Collectively, these observations suggest cross-regulation of iron-responsive and other metal-responsive gene networks in *S. oneidensis *MR-1.

### SO2426 binds in region of predicted recognition site upstream of *alcA*

Given the potential overlap in the response of *S. oneidensis *to iron and other metals, we chose to focus on the *S*. *oneidensis *siderophore biosynthesis operon in testing the interaction of two recombinant versions of the SO2426 protein with its predicted binding motif. The direct involvement of *so3030*-*3031*-*3032 *in the production of hydroxamate-type siderophores was recently demonstrated with deletion mutants within this gene cluster [43]. Induction of the *so3030*-*3031*-*3032 *operon in MR-1 cells under chromate challenge compared to unperturbed cells has been demonstrated using both transcriptomic and proteomic approaches [15,16]. In further studies with a *so2426 *deletion mutant under chromate challenge, the *so3030*-*3031*-*3032 *operon was significantly down-regulated [21,41]. These data, together with the predicted SO2426-binding motif upstream of *so3030*, suggest that SO2426 directly regulates siderophore production in strain MR-1 under conditions of chromate stress. We employed electrophoretic mobility shift assay (EMSA) to determine if the SO2426 protein was able to interact with the predicted binding sequence upstream of the *so3030*-*3031*-*3032 *operon.

**Figure 4:**
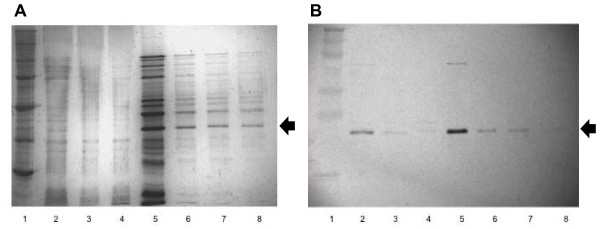
**Partial purification (A) and Western blot (B) verification of recombinant SO2426 protein**. Panel A, silver-stained gel of partial purification using a Ni-affinity column. Panel B, Western blot analysis performed in parallel with Anti-HisG Antibody (Invitrogen). Lanes: 1, MW markers; 2, cell lysate; 3, Wash 1; 4, Wash 2; 5-8, Elution Fractions 1-4. Recombinant SO2426 is denoted with an arrow.

Our previous 5' RACE studies demonstrated that the likely 5' terminus of SO2426 occurs at a methionine located at position 11 downstream from the originally annotated translation start [21]. Comparative sequence analysis of SO2426 with the CpxR and OmpR amino acid sequences from *V*. *cholerae *and *E. coli *showed that sequence homology between conserved receiver domains for these other well-characterized response regulators and SO2426 begins 13 amino acids downstream of the annotated start site for SO2426 [21]. This conservation is further observed for the *Shewanella *SO2426 orthologs (Figure 1). In order to test the functionality of the shorter version of SO2426, both the full-length annotated form (designated SO2426) and the "short" form beginning with M11 (designated SO2426sh), were expressed using the pTrcHis expression vector system, which incorporates an N-terminal six-histidine tag for affinity purification. The His-tagged proteins were expressed in *E. coli *and partially purified from crude cell extracts by Ni-affinity column purification (see Methods for details). Expression of the recombinant SO2426 protein was determined by SDS-PAGE (Figure 4A) and Western blotting (Figure 4B), which confirmed the presence of this protein within the expected size range of 26-27.4 kDa. Similar SDS-PAGE and immunoblotting results were obtained for the verification of recombinant SO2426sh expression (data not shown).

**Figure 5:**
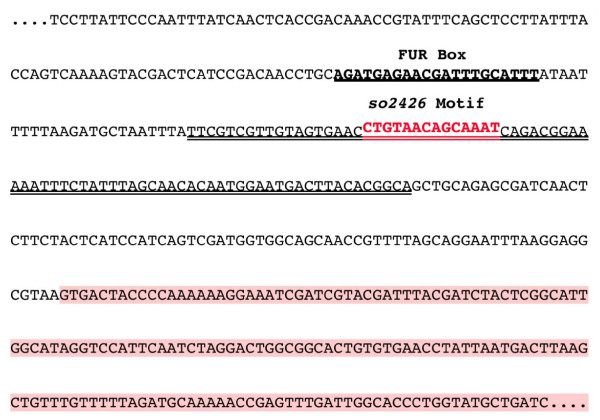
**Upstream nucleotide sequence of the siderophore biosynthesis *so3030*-*3031*-*3032 *operon**. The recognition site (Fur Box) for the ferric uptake regulator (single underline) and the predicted SO2426-binding motif (red type) are noted in the upstream region. A DNA probe for EMSA studies flanking the SO2426-binding motif was generated by PCR amplification (double underlined sequence). The 5' coding region of *so3030 *is highlighted in salmon.

**Figure 6:**
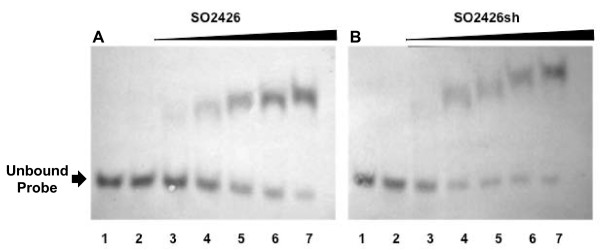
**Binding of recombinant SO2426 proteins to putative recognition site**. Electrophoretic mobility shift assays were performed to demonstrate binding of recombinant SO2426 (A) and SO2426sh (B) to the predicted SO2426 recognition motif upstream of the *so3030*-*3031*-*3032 *operon. Lanes: 1, DNA template only; 2, vector-only control *E. coli *cell lysate (15 μg); 3-7, increasing concentrations of either recombinant SO2426 or SO2426sh ranging from 0.6 to 3.0 μg in 0.6 μg increments. Each reaction mixture contained 95 ng of DIG-labeled DNA template. No binding was seen with an excess of vector-only control cell lysates (lane 2); whereas, a clear shift is seen with increasing amounts of either recombinant SO2426 or SO2426sh.

A digoxigenin-labeled DNA probe spanning the predicted SO2426-binding site motif upstream of the *so3030*-*3031*-*3032 *operon (Figure 5, double underlined region), but excluding the putative Fur box, was generated by PCR amplification and used as the DNA probe in measuring the DNA-binding activity of the partially purified recombinant SO2426 and SO2426sh proteins. Figure 6A shows that the DNA probe shifted upward in the presence of recombinant SO2426, with the shift becoming incrementally more enhanced as the protein concentration in the EMSA reaction mixture was increased. No shift was observed with protein extracts prepared from pTrcHis vector-only *E. coli *control strains, ruling out the possibility that the probe shift was due to non-specific binding of contaminating proteins. A comparable shift was observed for recombinant SO2426sh (Figure 6B), thus supporting our proposition that the actual 5' terminus of the SO2425 occurs at residue M11. Gel shift assays performed with additional DNA probes upstream of the *so3030*-*3031*-*3032 *operon as well as *so3036*, which also contains a putative SO2426 recognition sequence, showed a band-shift in the presence of recombinant SO2426 (data not shown). Although the primary focus in this study is the functional role of SO2426 in siderophore production, future studies will be necessary to analyze the interaction of SO2426 with additional recognition sites to further define its regulon.

### Siderophore production is deficient in a Δ*so2426 *mutant strain

Earlier physiological evidence for the role of SO2426 in siderophore production was obtained using liquid CAS assays in which relative siderophore production levels for the Δ*so2426 *mutant were compared to those for the wild-type MR-1 strain [21]. These studies demonstrated that the deletion mutant was markedly deficient in siderophore synthesis compared to the wild-type strain in LB medium supplemented with chromate [21]. LB medium constitutes a sufficient source of iron (~17 μM) [13]. Additionally, under iron-replete conditions, in which 50 μm FeCl_3 _was added to the medium, there was no change in siderophore levels in the Δ*so2426 *mutant. Conversely, siderophore production in the wild-type MR-1 strain returned to background levels in the presence of added iron [21].

**Figure 7:**
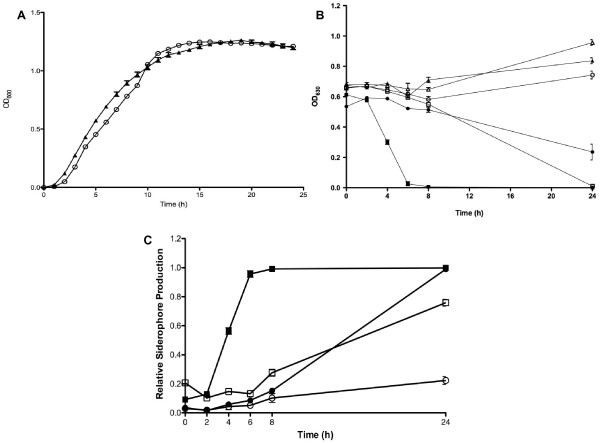
**Growth capacity and siderophore production by wild-type MR-1 and Δ*so2426 *strains in the presence of 2,2'-dipyridyl**. (A) Aerobic growth of wild-type MR-1 (closed triangles) and the Δ*so2426 *mutant (open circles) in LB supplemented with 80 μM of the Fe chelator 2,2'-dipyridyl. Cell growth was assessed for triplicate cultures and plotted as the mean OD_600 _± SEM. (B) Absorbance at 630 nm of CAS-treated samples in the absence (open symbols) and presence (closed symbols) of 2,2'-dipyridyl. Results are shown for wild-type MR-1 (squares), the Δ*so2426 *mutant (circles), and LB only (triangles). (C) Relative production of CAS-reactive siderophores by wild-type MR-1 (closed symbols) and the Δ*so2426 *mutant (open symbols) under aerobic growth conditions. 2,2'-dipyridyl (80 μM) was added to mid-log-phase (OD_600_, 0.6) MR-1 and Δ*so2426 *mutant cultures cultivated in LB broth, and relative siderophore synthesis was monitored over time using the CAS-based siderophore detection assay. The relative siderophore production was calculated by subtracting the supernatant A_630 _(absorbance at 630 nm) for the wild type or mutant from the control (uninoculated LB medium) and then determining the ratio of corrected supernatant A_630 _to control A_630_. Error bars represent the standard error of the mean for three replicate CAS measurements. Circles represent unamended LB cultures; squares represent iron-depleted cultures.

We expanded upon our previous physiological work by examining siderophore production under artificially imposed conditions of iron depletion. To achieve this, wild-type MR-1 and Δ*so2426 *mutant strains were allowed to grow in LB medium supplemented with 80 μM of the Fe chelator 2,2'-dipyridyl to simulate iron-limiting conditions. Other studies demonstrated that a 2,2'-dipyridyl concentration of ≤ 100 μM had a negligible effect, if any, on the growth rate of *S. oneidensis *MR-1 and certain mutant strains under aerobic conditions [14,43]. Similarly, we observed that MR-1 and the Δ*so2426 *mutant could grow aerobically at relatively normal rates in LB supplemented with 80 μM of 2,2'-dipyridyl (Figure 7A), indicating that environmental Fe was not scavenged below a critical Fe threshold necessary for growth. As shown in Figure 7B, the Δ*so2426 *mutant was unable to produce CAS-reactive siderophores at wild-type rates under aerobic growth conditions in the absence of 2,2'-dipyridyl. This deficiency was enhanced in the presence of iron chelator (Figure 7B). Relative siderophore production by wild-type MR-1 increased sharply, attaining a maximum level at the 6-h time point following exposure to 2,2'-dipyridyl (Figure 7C). At this time interval, we detected an 11-fold increase in the synthesis of CAS-reactive siderophores for MR-1 under iron depletion compared to MR-1 under iron-sufficient conditions (LB only). In the same 6-h time period, there was only a marginal elevation in siderophore production by the Δ*so2426 *mutant, which exhibited substantially reduced levels of siderophore production compared to MR-1 under iron depletion conditions (Figure 7C).

The impaired ability of the Δ*so2426 *mutant to produce siderophores during aerobic growth suggests that *so2426 *is a positive regulator of siderophore production in *S. oneidensis *MR-1. As noted earlier, several of the genes predicted to belong to the *so2426 *regulon also have Fur-binding motifs in their upstream regions. The likely molecular mechanism controlling iron homeostasis in *S*. *oneidensis *MR-1 involves Fur-mediated transcriptional repression, which includes down-regulation of *so2426 *expression under iron-replete conditions and derepression followed by SO2426-mediated transcriptional activation under iron-limited conditions. This may explain the residual siderophore production in the Δ*so2426 *mutant. It is also possible that an as-yet uncharacterized secondary mechanism for siderophore production exists in strain MR-1.

## Conclusions

SO2426 is annotated as a DNA-binding response regulator, but its specific function in *S*. *oneidensis *MR-1 was previously undefined. Using combined *in silico *motif prediction and *in vitro *binding assays along with physiological characterization, this report provides an important empirical step toward describing the SO2426 regulon. We initially identified a putative SO2426-binding consensus motif that consists of two conserved pentamers (5'-CAAAA-3') in tandem. Electrophoretic mobility shift assays demonstrated that recombinant SO2426 exhibits binding specificity with its predicted motif within the 5' regulatory region flanking a siderophore biosynthesis operon. A Δ*so2426 *mutant was unable to synthesize CAS-reactive siderophores at wild-type rates under iron limitation. Collectively, these data support a function for SO2426 as a positive regulator of siderophore-mediated iron acquisition in *S*. *oneidensis *MR-1.

In addition to exhibiting iron-responsive expression, the *so2426 *gene has been previously shown to be up-regulated in response to chromate stress [15,41]. The up-regulation of iron acquisition and iron storage systems in response to metal stress is not unique to *S. oneidensis*. In *Arthrobacter *sp. FB24, a number of proteins with putative functions in iron sequestration, such as Ferritin-Dps family proteins, as well as Reiske (2Fe-2S) domain proteins, showed increased abundance as a result of chromate stress [17]. Copper has been shown to disrupt Fe-S clusters in important enzymes in *E. coli *[44]. An *E. coli *strain defective in iron transport was also found to be more sensitive to chromium [19]. Exposure to manganese in *B. subtilis *resulted in altered intracellular iron pools with subsequent expression of Fur-regulated genes [45]. The reason for the up-regulation of iron-responsive genes is unclear. It has been speculated that metal ions such as chromate result in oxidative stress mediated through Fenton-type reactions with ferrous iron [18,46-48]. Up-regulation of iron storage proteins may help alleviate metal-induced oxidative damage by binding excess Fe and preventing its interaction with other metal ions. It is also apparent that proteins with Fe-S prosthetic groups as part of their active centers are primary targets of metal-induced damage. These processes undoubtedly disrupt intracellular iron homeostasis, leading to the up-regulation of iron acquisition and sequestration systems. The evidence provided here and in our previous work strongly points to an integral role of SO2426 in such iron control systems.

## Methods

### Bacterial strains, plasmids, and culture conditions

All strains and plasmids used in this study are described in Table 2. *E. coli *strains were cultured aerobically in Luria-Bertani (LB) [Difco, Detroit, MI] medium at 37°C with shaking. For recombinant *E. coli *strains, ampicillin was added to LB at a concentration of 50 μg/ml. *S. oneidensis *strains were grown aerobically in LB medium at 30°C with shaking at 200 RPM.

**Table 2:** Bacterial strains and plasmids used in this study

Bacterial Strains	Genotype	Source/Reference
*Shewanella oneidensis *MR-1	Wild type	ATCC 7005500 Lab stock
MR-1/Δ*so2426*	Deletion of *so2426 *locus	[21]
*E. coli *TOP10	Cloning and expression strain	Invitrogen
*E. coli *ER2508	Major proteinase-deficient strain	New England Biolabs
His-ER-2426-1-1	Expresses full-length SO2426 protein	This study
His-Top-26s-4	Expresses truncated SO2426 protein	This study
*E. coli *(pTOPO)	Vector-only control	Invitrogen

**Plasmids**		

pTrcHis-2426sh	*so2426sh *cloned in frame with N-terminal polyhistidine	This study
pTrcHis-2426	*so2426 *cloned in frame with N-terminal polyhistidine	This study

### SO2426 weight matrix development and identification of a putative SO2426 recognition site

MEME [30], MotifSampler [31], and Gibbs Recursive Sampler [32] were used to predict promoter recognition sequences potentially bound by SO2426. To facilitate motif searching, the time-series microarray expression profiles of the Δ*so2426 *relative to the parental strain were clustered using Hierarchical Clustering Explorer (HCE) [49]. During the clustering process, only genes with an expression value of at least ≥ 2-fold or ≤ 0.5-fold in one or more of 6 expression profiling time points were included in the analyses. As a result, a dataset of 841 genes was clustered based on the average linkage using Euclidean distance [21]. We extracted a sub-cluster comprising 46 similarly down-regulated genes throughout the 6 time points, and this dataset was used as the input data for putative SO2426 binding-site prediction. The consensus SO2426-binding sequence was predicted with MEME using the following parameters: (i) the motif width ranged from 6 to 50; (ii) the total number of sites in the *training set *where a single motif occurred was 3; and (iii) the sequence had 0 or 1 binding site. MAST [50] was used to scan the sequence database with the predicted MEME-derived motif. The Gibbs Recursive Sampler program was performed as described previously [12]. MotifSampler [31] was employed to confirm the consensus motif predicted using MEME and Gibbs Recursive Sampler. A sequence logo [51] was generated to graphically represent the sequence conservation of the predicted SO2426 recognition site.

### Sequence analysis of SO2426 orthologs

ClustalW [52] was used to perform a multiple sequence alignment of *Shewanella *SO2426 orthologs. Conserved signature residues in the receiver domain of response regulators were annotated based on reference [53]. The phylogenetic tree was constructed based on protein sequences using maximum parsimony method implemented in PAUP* version 4.0 Beta [54]. The bootstrap values were generated using maximum parsimony. The GenBank accession numbers are as follows: YP_734035.1, *Shewanella *sp. MR-4; YP_738119.1*Shewanella *sp. MR-7; YP_750834.1, *Shewanella frigidimarina *NCIMB 400; YP_869596.1, *Shewanella *sp. ANA-3; YP_927593.1, *Shewanella amazonensis *SB2B; YP_963447.1, *Shewanella *sp. W3-18-1; ZP_01705802.1, *Shewanella putrefaciens *200; YP_001050420.1, *Shewanella baltica *OS155; YP_001094061.1, *Shewanella loihica *PV-4; YP_001366502.1, *Shewanella baltica *OS185; YP_001474053.1, *Shewanella sediminis *HAW-EB3; YP_001502091.1, *Shewanella pealeana *ATCC 700345; YP_001554844.1, *Shewanella baltica *OS195; ZP_02156174.1, *Shewanella benthica *KT99; YP_001674438.1, *Shewanella halifaxensis *HAW-EB4; YP_001760668.1, *Shewanella woodyi *ATCC 51908; YP_002311920.1, *Shewanella piezotolerans *WP3; YP_002357973.1, *Shewanella baltica *OS223; NP_718016.1, *Shewanella oneidensis *MR-1; and YP_562912.1, *Shewanella denitrificans *OS217.

### Siderophore detection

The chrome azurol-S (CAS)-based assay for detection of siderophore production during cellular growth in liquid was performed as described elsewhere [21,55] with slight modifications in culture conditions. Overnight LB cultures of the Δ*so2426 *strain and the wild-type MR-1 strain were used to inoculate fresh LB broth and allowed to grow to mid-logarithmic phase (OD_600 _~ 0.6). The mid-log-phase cultures were amended with 50 μM FeCl_3_, 80 μM 2,2'-dipyridyl, or 0.3 mM K_2_CrO_4_. A control consisting of LB without amendment was prepared for each strain. The cultures were allowed to incubate for 24 h at 30°C with shaking. Aliquots were taken for CAS assay analysis at 0, 2, 4, 6, 8, and 24 h post amendment. Cell-free supernatants were mixed 1:1 with the CAS assay solution and equilibrated at room temperature for 2 h prior to reading the absorbance at 630 nm. The relative production of CAS-reactive siderophores was calculated as described [21] and reported as the average of three independent experiments.

### Expression and partial purification of recombinant SO2426 protein

Bacterial expression vectors were constructed by cloning the full-length SO2426 gene and a shortened form (SO2426sh) in frame with the N-terminal His-tag of pTrcHis (Invitrogen, Carlsbad, CA). Plasmids were transformed into *E. coli *TOP10 (Invitrogen, Carlsbad, CA) or *E. coli *ER2508 (New England Biolabs, Ipswich, MA) host cells. Transformants were selected on LB-ampicillin agar plates. Positive clones were verified by sequence analysis at the Purdue Genomics Core Facility.

Cells carrying the expression vectors were grown at 37°C in 100 ml of LB with 50 μg/ml ampicillin until a cell density of ~0.6 was attained. IPTG was added to a concentration of 1 mM, and the cultures were incubated for an additional 3 hours to induce expression of recombinant SO2426 proteins. Cells were harvested by centrifugation and washed in 1X TBS. Cell lysates were prepared by sonicating cell pellets in Guanidium Lysis Buffer, pH 7.8 (Invitrogen, Carlsbad, CA) containing 1X Complete-Mini Protease Inhibitor Cocktail (Roche Applied Science, Indianapolis, IN). The lysates were centrifuged at 6,000 RPM for 10 min to remove cell debris. His-tagged proteins were recovered from cell lysates using the ProBond Purification System (Invitrogen, Carlsbad, CA) under hybrid conditions as specified by the manufacturer's protocol. A total of eight 1 to 2-ml elution fractions were collected for each protein extract.

### Verification of SO2426 recombinant protein

Expression of His-tagged SO2426 and SO2426sh proteins in the elution fractions was verified by Western blot analysis using the Western Breeze Chromogenic Western Blot Immunodetection Kit (Invitrogen, Carlsbad, CA). His-tagged proteins were probed with an anti-HisG antibody (Invitrogen, Carlsbad, CA) with secondary detection using anti-mouse IgG-alkaline phosphatase antibody provided in the Western Breeze kit. Positive elution fractions were pooled and concentrated with YM-3 Centricon Centrifugal Filter Devices (Millipore, Billerica, MA). Concentrated fractions were dialyzed overnight at 4°C against TED buffer [20 mM Tris-Cl (pH 7.0), 150 mM NaCl, 0.1 mM EDTA, and 0.1 mM DTT] using mini dialysis tubes with a molecular weight cutoff of 8 kDa. Protein concentration was determined using a Nanodrop ND-1000 Spectrophotometer (Rockland, DE).

### Electrophoretic Mobility Shift Assay (EMSA)

A non-labeled DNA probe was first generated by PCR amplification of an 83-bp region upstream of *so3030 *using primers klh001 and klh004 (Table 3) and *S. oneidensis *MR-1 genomic DNA as a template. The probe sequence was verified by sequence analysis at the Purdue Genomics Core Facility. This PCR product was then used as the template in a PCR amplification reaction to generate a Digoxigenin-labeled DNA probe for use in EMSA. The reaction mixture consisted of 25 mM MgCl_2_, 1X Promega Go-Flexi *Taq *Polymerase buffer, a 1:6 ratio of dTTP:DIG-11-dUTP dNTP mix, 0.2 mM each of primers klh001 and klh004, 5.5 ng of the unlabeled PCR product as a template, and 10 U of *Taq *to 1 U Pfu cocktail in a final reaction volume of 50 μl. The PCR amplification cycle consisted of 95°C for 4 min and 30 cycles of 94°C for 1 min, 50°C for 30 sec, 72°C for 1 min, with a final extension step at 72°C for 5 min. Labelling efficiency was verified by Southern blot analysis using the DIG Nucleic Acid Detection Kit (Roche Applied Science, Indianapolis, IN) according to the manufacturer's protocol for colorimetric detection. The DIG-labeled DNA probe was quantified using a Nanodrop ND-1000 Spectrophotometer.

**Table 3:** Oligonucleotide primers used in this study

Primer	DNA sequence (5' → 3')	Reference or source
klh001	TTCGTCGTTGTAGTGAACC	This study
klh004	TGCCGTGTAAGTCATTCC	This study
2426F	ATGATATTGATTCTCGTTTGGT	This study
2426R	TTAAGCGCTAAAACTGTATCCTTG	This study
2426shF	ATGAGTAGAATACTGTTAGTCGAT	This study
2426shR	TTAAGCGCTAAAACTGTATCC	This study

EMSA was performed in 20-μl reaction volumes containing 0.5X EMSA buffer [5 mM Tris-Cl (pH 8.0), 75 mM KCl, 0.05 mM DTT, 0.05 mM EDTA, 6% glycerol], 5 mM MgCl_2_, 20 mM Acetyl-PO_4_, 0.2 μg/μl poly(dI:dC), 0.2 μg/μl BSA, and 95 ng DIG-labeled DNA probe. Protein was added in concentrations ranging from 0.6 to 3.0 μg in increments of 0.6 μg. Reactions were incubated at 16°C for 30 min. NP-40 was added to each reaction mixture at a concentration of 0.1% prior to separation on a pre-run 5% polyacrylamide gel. Gels were stained with SYBR green and then transferred onto Hybond N+ (Amersham Biosciences, Piscataway, NJ). Probing and detection of DIG-labeled DNA was performed with the DIG Nucleic Acid Detection Kit according to the manufacturer's protocol for colorimetric detection.

## Authors' contributions

KLH carried out the expression and partial purification of the recombinant SO2426 and SO2426sh proteins, performed electrophoretic mobility shift assays and siderophore production measurements, and wrote the majority of the manuscript. XFW generated the multiple sequence alignment and phylogenetic tree for SO2426 orthologs in *Shewanella*, identified the predicted recognition site for SO2426 binding, and contributed to the production of the manuscript. WW constructed the vectors for recombinant SO2426 and SO2426sh expression. DKT conceived the study, helped to supervise the experiments, and participated in the writing of the manuscript. All authors read and approved the final manuscript.
